# Development of Interpolyelectrolyte Complex Based on Chitosan and Carboxymethylcellulose for Stabilizing Sandy Soil and Stimulating Vegetation of Scots Pine (*Pinus sylvestris* L.)

**DOI:** 10.3390/polym16162373

**Published:** 2024-08-22

**Authors:** Nazira Berikbol, Alexey Klivenko, Vadim Markin, Lazzyat Orazzhanova, Gulnur Yelemessova, Zhanar Kassymova

**Affiliations:** 1 Department of Chemistry and Ecology, Research School of Physical and Chemical Sciences, Shakarim University of Semey, Semey 071412, Kazakhstan; nazira_berikbolova@mail.ru (N.B.); alexeyklivenko@mail.ru (A.K.); lazzyat.orazzhanova.70@mail.ru (L.O.); kussainova_g91@mail.ru (G.Y.); 2 Department of Organic Chemistry, Institute of Chemistry and Pharmaceutical Technologies, Altai State University, Barnaul 656049, Russia; markin@chemwood.asu.ru

**Keywords:** interpolyelectrolyte complex, natural polymers, chitosan, sodium carboxymethylcellulose, sandy soils, soil erosion, scots pine

## Abstract

The issue of water and wind erosion of soil remains critically important. Polymeric materials offer a promising solution to this problem. In this study, we prepared and applied an interpolyelectrolyte complex (IPEC) composed of the biopolymers chitosan and sodium carboxymethyl cellulose (Na-CMC) for the structuring of forest sandy soils and the enhancement of the pre-sowing treatment of Scots pine (*Pinus sylvestris* L.) seeds. A nonstoichiometric IPEC [Chitosan]:[Na-CMC] = [3:7] was synthesized, and its composition was determined using gravimetry, turbidimetry, and rheoviscosimetry methods. Soil surface treatment with IPEC involved the sequential application of a chitosan polycation (0.006% *w*/*w*) and Na-CMC polyanion (0.02% *w*/*w*) relative to the air-dry soil weight. The prepared IPEC increased soil moisture by 77%, extended water retention time by sixfold, doubled the content of agronomically valuable soil fractions > 0.25 mm, enhanced soil resistance to water erosion by 64% and wind erosion by 81%, and improved the mechanical strength of the soil-polymer crust by 17.5 times. Additionally, IPEC application resulted in slight increases in the content of humus, mobile potassium, mobile phosphorus, ammonium nitrogen, and mineral salts in the soil while maintaining soil solution pH stability and significantly increasing nitrate nitrogen levels. The novel application technologies of biopolymers and IPEC led to a 16–25% improvement in Scots pine seed germination and seedling growth metrics.

## 1. Introduction

Forest natural resources possess economic and ecological value and contribute socially to human development [1,2]. They provide timber, medicinal, technical, and food raw materials, as well as perform ecosystem functions such as soil protection from erosion, oxygen production, climate stabilization, and biodiversity preservation [3,4]. However, the stable role of forest ecosystems is being diminished by persistent natural and anthropogenic degradation, which is associated with climate aridization, deforestation, wildfires, intensive agriculture, overgrazing, air pollution, the presence of pests, and fungal infestations [5,6]. These harsh forest-growing conditions complicate forest regeneration and afforestation in Kazakhstan and other countries around the world. Currently, forest cover occupies no more than 5% of Kazakhstan’s territory, with coniferous forests comprising 0.7% of the country’s forested area [7,8]. Special attention is given to the restoration of the relict ribbon forests near Semey (Abay region, Kazakhstan), for the protection of which the State Forest Nature Reserve (SFNR) “Semey Ormany” has been operating since 2003 [9]. Annually, approximately 67 fires occur in relict pine forests, destroying about 5700 hectares. In recent years, the highest rate of forest loss due to wildfires was recorded in 2023, amounting to over 60,000 hectares of forested areas [10,11]. 

To enhance the efficiency of reforestation after a wildfire, modern technologies for growing planting materials are essential. The quality of the planting material depends on the quality of the seeds and soil fertility. To ensure seed resistance to pests, fungal diseases, and adverse environmental conditions before sowing, various chemical plant protection agents are used [12]. However, the use of pesticides and stimulants in the process of growing pine seedlings is accompanied by certain negative consequences, such as non-selectivity [13], high toxicity [14,15], and accumulation of chemicals [16,17], leading to teratogenesis in pine seedlings [18,19]. Additionally, a widely discussed global environmental issue in post-wildfire reforestation is soil erosion [10,20,21], as the physical-chemical and hydrothermal properties of the topsoil layer are altered [22]. This results in the formation of a non-water-resistant structure with a high content of soil particles less than 0.25 mm, low humus content, weak cohesion of soil particles, low moisture capacity, and poor water retention ability [23,24]. The application of biological methods, engineering methods, and rational agrotechnologies to improve the structural condition of soil requires significant time, labor, and economic resources. These methods demonstrate a limited effect duration and may have potential environmental consequences, necessitating regular maintenance and monitoring [25,26]. Currently, among the chemical methods for soil structure formation, synthetic and natural polymeric materials are of the greatest interest due to their commercial availability, diverse chemical composition and spatial structure, water solubility, and chemical stability over a wide range of pH, ionic strength, and temperature. The literature widely features publications dedicated to the most common synthetic polyacrylamide (PAM) soil conditioners, which are actively used on an industrial scale. In the paper of Panova I.G. et al. [27], the mechanisms and effects of the stabilizing action of the anionic polyelectrolyte PAM and its interpolyelectrolyte complexes (IPECs) on agricultural soils are described in terms of polymer consumption rates, polymer properties, and substrate characteristics. At the same time, the authors note the disadvantages of PAM, such as neurotoxic, carcinogenic, and mutagenic effects when inhaled or in contact with the skin, with more than 0.05% of acrylamide by weight of the commercial product, as well as its accumulation in soils and wastewater, and the reduction of the structuring effect during the hydrolysis of water-soluble PAM. 

Despite the widespread use of polymeric materials for soil quality improvement, there are relatively few examples of their use in enhancing the properties of forest soils. Examples of the applications of polyacrylamide and polyacrylates for forest soil stabilization are presented in Table 1.

Environmentally and economically promising alternatives to synthetic polymeric soil conditioners, as well as to stimulants and pesticides for the development and protection of planting material, are biopolymers and their IPECs [26,33,34,35]. The use of natural polymers reduces their toxic impact on the environment, as they are biodegradable [36,37,38] and participate in the organic carbon cycle, providing the “soil-plant” system with nutrients.

**Figure 1:**
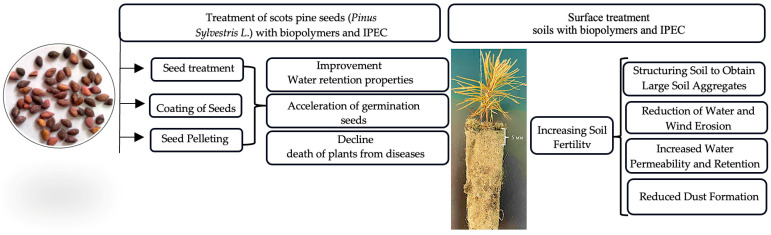
Effects of seed and soil treatment with biopolymers and interpolyelectrolyte complex (IPEC).

Figure 1 presents the methods of using biopolymers and IPECs for reforestation [39,40,41,42,43,44,45,46,47,48,49,50]. 

Among the many biopolymers used for agroecological and reclamation purposes, chitosan and sodium carboxymethylcellulose (Na-CMC) are of particular interest due to their valuable chemical composition and biological properties [51,52].

**Figure 2:**
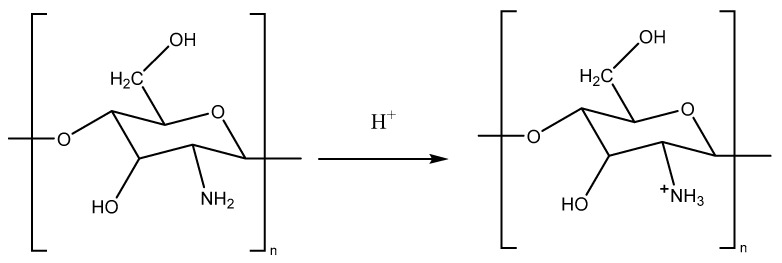
Protonation of chitosan in acidic medium.

Chitosan is an aminopolysaccharide, 2-amino-2-deoxy-β-D-glucan, which is a deacetylated derivative of chitin. Due to the protonation of free amino groups in aqueous acid solutions, chitosan acts as a polycation and forms IPEC with anionic compounds and surfaces (Figure 2) [53,54]. 

The biocompatibility, bioactivity, biodegradability, sorption capacity, and non-toxicity of chitosan are influenced by the degree of polymerization, degree of deacetylation, ratio and pattern of acetylated and deacetylated residues in the polymer chain, and molecular weight [55,56]. Na-CMC is the sodium salt of a simple ether of cellulose and glycolic acid, in which the hydroxyl groups of the monomer are partially substituted by carboxymethyl groups. This modification imparts an anionic character to Na-CMC molecules [57]. The biological and physicochemical properties of Na-CMC depend on the degree of polymerization and uniformity of carboxymethyl substitution. Due to its good solubility in water and ability to form highly viscous solutions, Na-CMC forms biodegradable, strong, flexible, hygroscopic, and transparent films [58,59].

**Figure 3:**
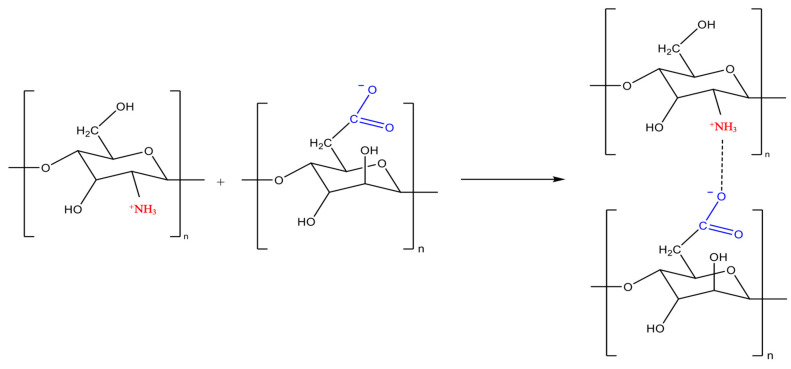
Scheme of IPEC Formation from chitosan and sodium carboxymethylcellulose (Na-CMC).

Electrostatic interactions between the positively charged amino groups (NH_3_^+^) of chitosan and the negatively charged carboxylate groups (COO^−^) of Na-CMC lead to the formation of IPEC (Figure 3) [60,61].

The advantages of IPECs include their ability to change phase states in response to various external factors (pH, ionic strength, and temperature) and ease of synthesis. IPEC can be obtained using several methods: (1) mixing solutions of interacting polymers in a common solvent to form a complex; (2) mixing polymers at the interface of two immiscible liquids; and (3) matrix polymerization [62,63,64,65].

Depending on the conditions of the interpolyelectrolyte reaction, the formation of stoichiometric (s-IPEC) or nonstoichiometric (n-IPEC) complexes is possible. Poorly soluble and insoluble s-IPECs typically form at a molar ratio of 1:1 between the monomeric units of the interacting polyelectrolytes [66]. In n-IPECs, the ratio of monomer units does not correspond to whole numbers, which leads to unique structuring properties, such as thermostability, chemical resistance, biocompatibility, adjustable mechanical strength, electro-optical properties, permeability, and selectivity. In the paper by I.G. Panova et al. [67], the superior stabilizing effect of n-IPEC with a threefold excess of cationic poly (N, N′-diallyl-N, N′-dimethylammonium chloride (PDADMAC) groups compared to s-IPEC from potassium humates and PDADMAC at the same IPEC application rate is noted.

In the formation of a polymer coating for soil structuring, the adhesive properties of the polymers and their high binding capacity are used, owing to the super-equivalent adsorption by negatively charged soil particles, as well as interactions between polymer molecules forming intermolecular and chemical bonds in the IPEC [52,68].

Between IPEC and soil particles, covalent bonds, as well as Van der Waals forces, can develop, which promote intermolecular cohesion between soil particles, stabilize, and increase their mass [51,69]. The formation of covalent bonds between IPEC and soil particles is influenced by the number and distribution of functional groups (-COOH, -OH, and -NH_2_) in the interacting polymers.

The introduction of biopolymers and the formation of IPEC directly in the soil involves the following stages: (1) separate preparation of dilute polyelectrolyte solutions; (2) sequential introduction of the obtained solutions into the soil by spraying onto the soil surface; (3) spreading of the polymer adhesive over the soil surface and its wetting; (4) establishment of adhesive contact, depending on the macromolecular properties of the adhesive and the processes of adsorption and diffusion in the surface soil layer; (5) formation of the chemical and physical structure of the “adhesive-surface layer” system [70,71,72].

On the soil surface, IPEC forms a protective crust and cloddy soil structures that are resistant to wind and water erosion. This form of protection positively affects soil pore distribution, improves the soil’s water-physical properties, enhances biological activity, significantly increases the rate of water infiltration into the soil, and promotes rapid drying and heating of the soil surface [73,74].

In our previous publications [52,53,55,72], we presented results demonstrating the effective use of IPECs created from biopolymer pairs such as chitosan-Na-CMC, chitosan-Na-alginate, and chitosan-PAA for structuring urban and agricultural soils in Semey City (East Kazakhstan), as well as for biostimulating the yield of vegetable crops. The distinction of the current study lies in the investigation of the application of IPECs to forest soils and coniferous plants, specifically through the pre-sowing treatment of Scots pine (*Pinus sylvestris* L.) seeds, as well as the treatment of sandy pine soils to stabilize them against water and wind erosion.

## 2. Materials and Methods

### 2.1. Objects and Materials

The object of the laboratory study was the soil of a pine forest located in the Irtysh Depression on the southeastern edge of the West Siberian Plain, on the right bank of the Irtysh River (50°25′0″ N, 80°18′0″ E). The region’s climate is continental and arid. The average annual air temperature ranges from −0.66 to 9.54 °C, with a warm period from April to September lasting 200 days. The annual precipitation is 250–300 mm. Strong winds blow throughout the year, mainly from the west and southwest. The average annual wind speed in most areas is 2.5–4.5 m/s [75]. 

Soil sampling was conducted in the autumn of 2021 in flat and hilly areas of sandy forest regions near Semey City. Soil samples were collected from the upper horizon to a depth of 25 cm. 

Chitosan from crab shells and Na-CMC were purchased from Sigma Aldrich (Saint-Louis, MO, USA) and used without further purification. The molecular weights (MM) of the polymers and the degree of deacetylation (DS) of chitosan were determined using the methods described in the paper by Klivenko A.N. et al. [55]. The degree of substitution and content of carboxymethyl groups (CMG) in Na-CMC were determined by potentiometric titration, as described by Mossa I. et al. [76] and Markin V.I. et al. [77]. 

The preparation of the polymer solutions is described in a paper by Klivenko A.N. et al. [55]. To prepare individual biopolymer solutions with a concentration of 0.01 mol/L, 2.42 g of Na-CMC was dissolved in distilled water, and 1.61 g of chitosan was dissolved in 3% citric acid C_6_H_8_O_7_ (Meryer, Shanghai, China). Citric acid is a biotic solvent that participates in the metabolic processes of living organisms. The polymer solutions were mixed in a shaker (Stegler, Shanghai, China) for 24 h and brought to a volume of 1 L. The pH of the resulting polymer solutions was measured using a pH meter (Mettler Toledo, 8603, Greifensee, Switzerland). The solubility (S) of the polymers was determined using a gravimetric method.

Seeds of Scots pine (*Pinus sylvestris* L.) were used for the vegetation experiment, as this subspecies of pine is the main forest-forming tree species in Semey pine forests. Seeds of the first category of the 2021 stand yield were collected in the Beskaragai district of the Kanonersky branch of the National Reserve “Semey Ormany”, with an average mass of 1000 seeds being 9.7 ± 1.8 g and a purity of 93%. Seed quality was determined as described in the study by Tsitson T.K. [78]. 

### 2.2. Synthesis of IPEC

IPEC was synthesized by direct mixing, adding a polycation to a polyanion, and preparing equimolar solutions of oppositely charged polyelectrolytes [chitosan]:[Na-CMC] in molar ratios of [0:10], [1:9], [2:8], [3:7], [4:6], [5:5], [6:4], [7:3], [8:2], [9:1], and [10:0]. Equimolar polymer solutions were mixed in a shaker (Stegler HS, China) for 2 h at a temperature of 25 °C.

### 2.3. Gravimetric Analysis of Complex Formation

The resulting IPEC precipitates were centrifuged in a laboratory centrifuge (Centrifuge CM-6M Elmi, Riga, Latvia) at 3500 rpm for 15 min. The supernatant was then decanted, and the wet precipitates were weighed. The precipitates were dried using a freeze dryer (Freeze dryer Scientz-12, Ningbo, China) for 5 days at 41 °C to a constant mass and weighed on analytical scales (Mettler Toledo ML 204, Greifensee, Switzerland) with an accuracy of 0.0001 g. Based on the obtained data, a graph of the dependence of the precipitate mass on the molar composition of the complex was constructed.

### 2.4. Turbidimetric Analysis of Complex Formation 

After shaking, the IPEC solutions were transferred to a cuvette with a layer thickness of 1 cm, and the optical density at a wavelength of 400 nm was measured on a spectrophotometer Specord 210 plus (AnalytikJena, Jena, Germany). Based on the obtained data, a graph of the dependence of the optical density on the molar composition of the reaction mixture was constructed.

### 2.5. Rheoviscometric Analysis of Complex Formation

The rheological properties of the IPEC were studied using a rotational viscometer (HAAKE VT550, Thermo Scientific, Waltham, MA, USA). Measurements were conducted in the CR mode. The stationary flow curve was recorded at shear rates ranging from 50 to 1000 s^−2^. Measurements and data processing were carried out using RheoWin 4.0 software, as described in the practical guide by Schramm G. [79]. 

### 2.6. Soil Sampling and Preparation

Soil sampling and preparation were conducted according to the method in the papers Kim, H. Tan. [80].

### 2.7. Determination of Agrochemical Soil Indicators

The determination of hygroscopic moisture, actual and exchange acidity, and the content of mobile phosphorus, humus, and nitrogen were carried out according to the methodology described in the paper by Orazzhanova, L.K. et al. [53]. The cationic and anionic compositions were determined according to the methodology described in the paper by Akimzhanova K.G. et al. [81].

### 2.8. Determination of the Mechanical Composition of the Soil

Soil structure analysis was performed using the dry and wet sieving method, as described in the paper by Orazzhanova, L.K. et al. [53]. Based on the dry sieving data, the structural coefficient (K_str_) was calculated using Equation (1):

(1) $$K_{str}=\frac{\sum \left(10-0.25\;mm\right)}{\sum \left(10\;mm,<0.25\;mm\right)},$$

where the numerator represents the mass of soil aggregates between 10 and 0.25 mm, and the denominator represents the mass of soil aggregates greater than 10 mm and less than 0.25 mm.

Based on the dry sieving data, the cloddiness of the soil (K), an indicator of the potential wind resistance of the soil surface layer, was also calculated using Formula (2):

(2) $$K=\frac{\sum \left(>1\;mm\right)}{M},$$

where the numerator represents the mass of aggregates larger than 1 mm, and M—mass of the soil sample taken for analysis, in grams.

The criterion for the water stability of soil aggregates (W, %) was calculated using Formula (3):

(3) $$W=\left(\frac{A_{1}\;}{A_{2}\;}\;\right)\times 100\%,$$

where A_1_ and A_2_ are the masses of aggregates between 1 and 0.25 mm as determined by wet and dry sieving, respectively, in grams.

### 2.9. Microscopic Study of the Structure of Soil Aggregates after Treatment with Water and Application of IPEC

Microphotographs of the soil aggregates were obtained using a low-vacuum analytical scanning electron microscope (SEM JSM-6390LV, Jeol, Tokyo, Japan).

### 2.10. Modeling the Stability of Soil Aggregates against Wind and Water Erosion in Laboratory Conditions

To study the soil’s resistance to wind and water erosion, 10 cm diameter Petri dishes were filled with 160 g of soil fraction 0.50–0.25 mm from dry sieving, taken from the upper layer of pine sands. Laboratory experiments were conducted using the following variants in three replicates:

Soil treated with distilled water (control).

Soil treated with acid-soluble chitosan (at a rate of 0.02% polymer by weight of air-dry soil).

Soil treated with water-soluble Na-CMC (0.03% polymer by weight of air-dry soil).

Soil treated with IPEC (0.006% chitosan and 0.02% Na-CMC polymer by the mass of air-dry soil with a molar ratio of [Chitosan]:[Na-CMC] = 3:7). The molar ratio was selected based on the results of our previous studies on the optimal composition of IPEC.

Soil treatment with IPEC was conducted using the two-solution method described in our previous works [52,53]. The process involved the sequential application of diluted polymer solutions to the soil surface using a sprayer. First, a 0.01 M chitosan solution was evenly sprayed onto the soil surface. After the complete absorption of the first polymer, a 0.01 M solution of the second polymer, Na-CMC, was evenly sprayed.

**Figure 4:**
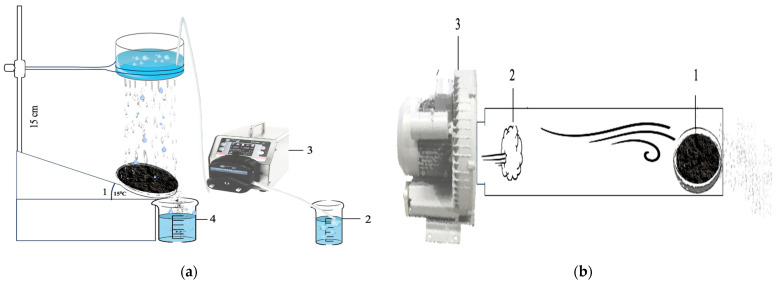
(**a**) Laboratory setup for modeling water erosion (**b**) Laboratory setup for modeling wind erosion.

For the water erosion study, Petri dishes with five-day dried soil-polymer structures were placed at a 15° angle (Figure 4a) (1) and sprayed with 500 mL of distilled water (Figure 4a) (2) at room temperature using a sprinkler system. Water was supplied using a BT100 peristaltic dosing pump (ADS-Lab, Irkutsk, Russia) (Figure 4a) (3). Runoff water was collected in glass containers, and turbidity was compared across variants (Figure 4a) (4). Simultaneously, the soil residues in the Petri dishes were dried in a drying oven (58/350 LPF AB UMEGA, SnolTherm, Utena, Lithuania) at 100 °C to a constant mass. The dried soil samples were reweighed to determine the loss from water runoff. 

For the wind erosion study, Petri dishes with five-day dried soil-polymer films (Figure 4b) (1) were placed horizontally and exposed to air at a speed of 12 m/s (Figure 4b) (2) for 1 min using a duct fan (VKK-200, Teplomash, Saint-Petersburg, Russia) (Figure 4b) (3). We developed laboratory setups for modeling wind and water erosion, as presented in Figure 4.

The resistance (R, %) of the soil to water and wind erosion was calculated using Formula (4):

(4) $$R=\left(\frac{m\;}{M\;}\right)\times 100\%,\;$$

where m is the mass of the soil that remained after the test in grams, and M is the initial mass of the soil sample taken for the test in grams [53].

### 2.11. Determination of IPEC’s Effect on Soil Moisture Retention

In soils treated with biopolymers and IPEC, the moisture content was measured with an accuracy of 0.01% until a constant mass was reached using a moisture analyzer (MX-50 A&D Weighing, A&D company, Tokyo, Japan).

### 2.12. Determination of Soil Mechanical Strength

The mechanical strength of the soil-polymer films was studied using a TAXT texture analyzer (Stable Micro Systems, Godalming, Surrey, UK). For this, the sample was placed on the device and tested in compression mode at a speed of 0.01 mm/sec using the P/5S probe, which is a needle with a 5 mm diameter ball at the end.

Based on the obtained data, a graph of the sample deformation versus applied stress was constructed. Young’s modulus was determined as the slope of the initial part of the deformation versus stress curve. The total stress was defined as the maximum value of the first peak on the stress-deformation curve. All samples were tested at least five times, and the results were averaged.

### 2.13. Study of IPEC Bioactivity Using Vegetation Method

Experiments on the influence of polymers and their IPEC on seed germination and seedling development of pine were conducted in the laboratory of the National Center for “Radioecological Research” at Shakarim University of Semey and in the forest seed complex of SFNR “Semey Ormany”.

During the experiment, 100 pine seeds were placed on filter paper in Petri dishes, divided into 4 sectors of 25 seeds each, ensuring that they did not touch each other. Before polymer treatment, the seeds were disinfected with a 0.05% KMnO_4_ solution for 30 min. Then, they were soaked in polymer solutions with a concentration of 0.01 mol/L and IPEC with a molar ratio of [3:7] for 60 min.

Observations of seed development were carried out over 15 days according to the methodology described in the paper by Kapar B. et al. [82]. Germination energy was determined on the seventh day, and uniformity was determined by daily counting of germinated seeds and germination rate, as well as measuring the average root length and weighing their mass after 15 days.

The germination rate is the percentage ratio of the number of germinated seeds to the total number of seeds tested. Germination (G, %) was calculated using the following Formula (5):

(5) $$G=\frac{a_{1}}{a_{2}}\times 100,\;$$

where a_1_ is the number of germinated seeds in 15 days, and a_2_ is the total number of seeds and pieces.

Germination energy determines the percentage of seeds that germinate within the first three days relative to the total number of sown seeds. The calculation of germination energy (E, %) was performed according to Formula (6):

(6) $$E=\frac{N}{a_{2}}\times 100,$$

where N is the number of seeds germinated within the first 7 days.

The germination rate (C, seeds/day) is determined as the average number of seeds germinating each day. The calculation of germination rate was conducted using Formula (7):

(7) $$C=a+\frac{b}{2}\ldots \ldots \frac{c}{15},$$

where a, b, c—number of seeds sprouted in 1, 2 … 15 days; 1, 2 … 15—number of days.

The uniformity of germination represents the average percentage of seeds germinated per day. Uniformity (U, %/day) was calculated using Formula (8):

(8) $$U=\frac{G}{D}$$

where G is the germination rate (%), and D is the number of germination days.

Polymers and IPEC were used to treat the soil surface during the cultivation of Scots pine (*Pinus sylvestris* L.) seedlings as biostimulants for plant development and soil structure formation. Seeds were grown in specially designed “HIKOV-120 SS” trays with dimensions of 352 mm × 216 mm × 110 mm, containing 40 cells, which are most suitable for use in arid regions with a sharp continental climate.

Studies on the morphological state of pine seedlings were conducted in four variants with three replications each: 1. control (soil treated with distilled water), 2. soil treated with acid-soluble chitosan, 3. soil treated with water-soluble Na-CMC, 4. soil treated with IPEC.

Every 15 days for three months, the following biometric characteristics of the seedlings were determined: height, diameter of the above-ground part, needle length, root length, and total phytomass.

## 3. Results and Discussions

In this study, the biopolymers chitosan and Na-CMC, as well as their IPEC, were applied for the first time as soil structuring agents in forest soils to enhance the growth activity of pine seedlings.

Physico-chemical characteristics of the polymers used are shown in Table S1. 

To achieve this goal, the optimal composition of the IPEC polymer formulation was selected. When mixing solutions of individual biopolymers with equimolar concentrations, turbidity of the solution and precipitation of IPEC were observed. The obtained precipitates were white, had an airy structure resembling medical cotton, felt like a dense gel-like mixture upon contact, and were odorless. The dried IPEC precipitates resembled a cluster of very thin threads and clumps (Figure S1).

**Figure 5:**
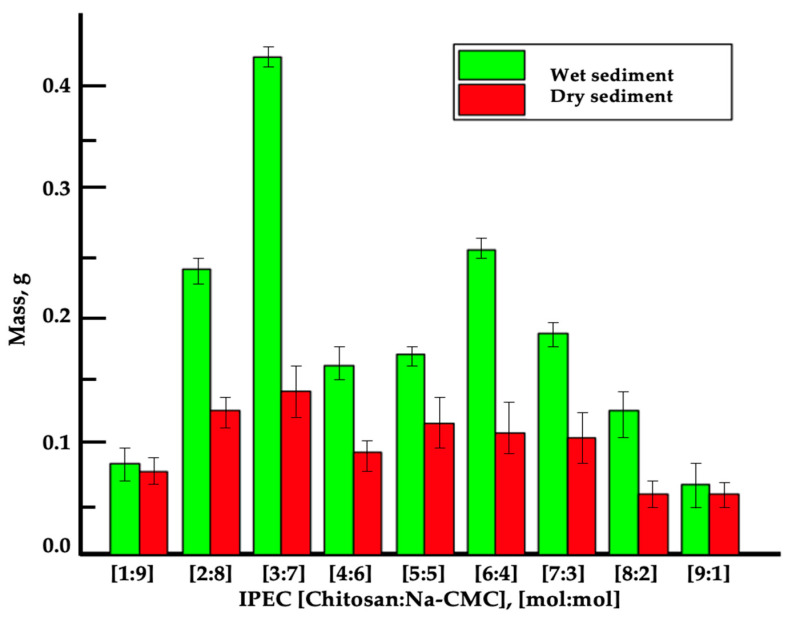
Masses of wet and dry IPEC precipitate with various molar compositions.

The quantitative yield of the synthesized IPEC at various molar ratios of the components was determined gravimetrically. The wet IPEC precipitates were weighed after centrifugation, and the dry IPEC precipitates were weighed after lyophilization. The masses of the resulting precipitates are presented in Figure 5. 

The maximum yield of wet IPEC product—0.4530 g and dry product—0.1151 g was observed at a polymer molar ratio of [3:7]. This ratio was chosen as optimal, and the obtained IPEC was applied to seed and soil treatment in the laboratory and nursery. The formation of water-insoluble ns-IPEC is likely due to the high hydrophobicity of chitosan and the low geometric matching of charge locations on the macromolecules. The use of nonstoichiometric IPEC in soil improves the aggregation process of mechanical particles compared to traditional methods. The free functional groups of IPEC provide additional adhesion to particles of various origins on the surface, ultimately enhancing the strength of the formed soil clumps.

**Figure 6:**
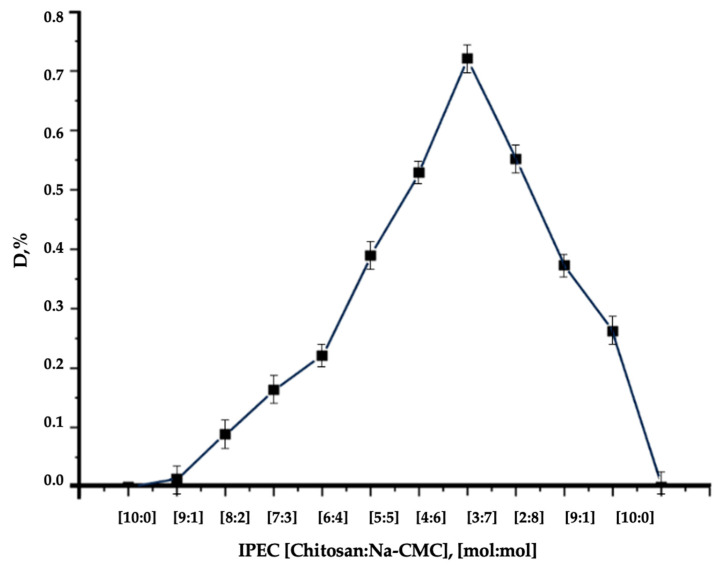
Dependence of the optical density of the solution on the composition of the IPEC reaction medium.

To further detail the composition of IPEC, complex formation was studied using the turbidimetric method. Turbidimetry showed that the solution had the lowest light transmittance (D = 0.75) at the selected optimal molar ratio of components in IPEC [3:7] (Figure 6) [83].

In our previous studies, the optimal composition of IPEC [3:7] was also determined using IR spectroscopy [52,55].

Studying the rheological properties of polymer solutions is necessary to predict their penetration depth into the soil and their solidification as a soil-polymer film, as rheological properties are sensitive to changes in the molecular structure of biopolymers and IPEC [84,85]. The rheological behavior of polymer systems is determined by their formation mechanism, with particular importance placed on the environment, spatial arrangement, and interaction of functional groups within the polymer matrix.

**Figure 7:**
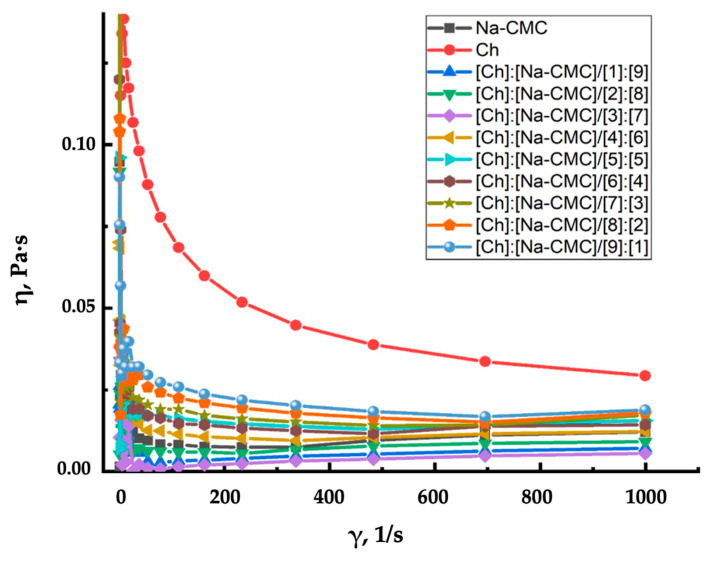
Dependence of the viscosity of the polymer solutions and IPEC of different molar compositions on their shear rate.

Figure 7 depicts the viscosity dependence of the polymer and IPEC solutions of varying molar compositions on their shear rate. Curve (1) represents the flow behavior of chitosan [10:0], which is characteristic of pseudoplastic fluids, where shear stress increases while viscosity decreases with increasing shear rate. The other studied systems (2) exhibit dilatant flow, where viscosity increases with increasing shear deformation rate [55]. Generally, the non-Newtonian fluids studied are flowable under quiescent conditions but exhibit solid-like properties under sudden impact. When interacting with the solid soil surface, the polymer and IPEC solutions form a protective soil-polymer film against the destructive effects of wind and water. 

**Figure 8:**
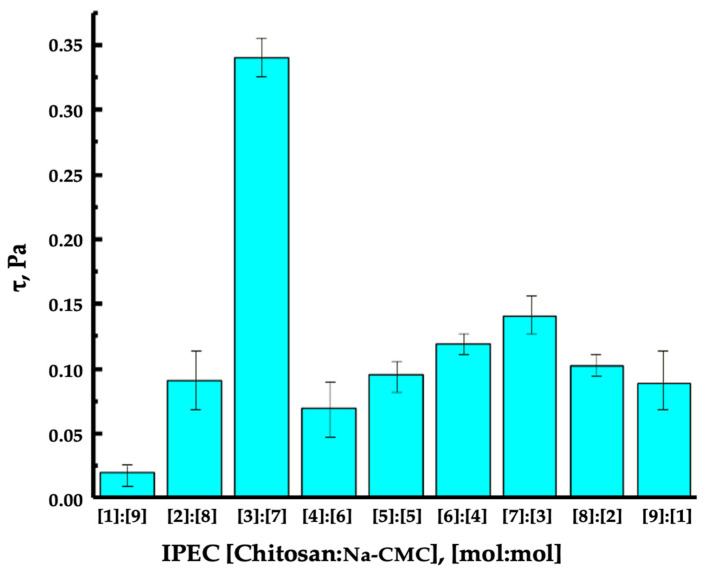
Shear stress curves of IPEC solutions of different molar compositions [Chitosan]:[Na-CMC].

Rheological studies also allow determining the shear stress of IPEC solutions of different molar compositions. As can be seen from Figure 8, at the molar ratio of polymers in IPEC [Chitosan]:[Na-CMC] [3:7], the highest shear stress is observed, which indicates the least displacement and the highest viscosity of the obtained IPEC.

The next stage of the study was to examine the agrochemical indicators and mechanical composition of pine forest sands before and after treatment with IPEC [Chitosan]:[Na-CMC] [3:7] (Table 2).

The obtained data indicate that the upper horizons (0–25 cm) of forest sandy soils are characterized by a sandy mechanical composition, low humus content, nearly neutral actual and exchange acidity, low levels of mobile nitrogen and phosphorus, medium levels of mobile potassium, low water retention capacity, and low soil solution salinity.

The content of macro- and mesoelements, such as P_2_O_5_, K_2_O, SO_4_^2−^, and Cl^−^, slightly increased in the experimental variants with the application of IPEC. The application of IPEC influences various biochemical transformations in soil. Under the influence of IPEC, microorganisms release nitrogen, phosphorus, potassium, and other microelements from the organic matter. The pH of the soil solution also slightly decreased after the application of IPEC. The significant increase in NO_3_^−^ content may be associated with the presence of amino groups in the macromolecules of chitosan, which serve as a reservoir of nitrate nitrogen through the processes of ammonification and nitrification in the soil (NH_2_ → NH_4_^+^ → NO_2_^−^ → NO_3_^−^) [53]. IPECs can form porous structures and a surface crust on the soil, binding soil particles that retain water, preventing the leaching of elements and ensuring their gradual release into the soil [27]. The amino groups (NH3⁺) of chitosan can bind negatively charged ions, such as nitrates (NO_3_^−^) and chlorides (Cl^−^), through electrostatic interactions and chelation. This helps to retain nitrate nitrogen in the soil, increasing nitrogen availability for plants and stimulating their growth and development. The obtained data align with numerous studies showing that the application of polymeric soil structure formers increases agricultural crop yields by 10–40% [86,87]. The lower tendency of chloride ions (Cl^−^) to engage in electrostatic interactions and complexation with polymers and IPEC is due to their simple structure, small size, low polarizability, weak interactions with positively charged soil particles, competition with other ions, and chemical inertness. These factors make chloride ions more mobile in the soil and more prone to leaching [88].

The results of the quantitative ratios of the mechanical element fractions in the soil samples are presented in Table 3.

The aggregate composition of the soil structure was determined using the structural coefficient (K_str_) and cloddiness coefficient (K). The results of the dry sieving of the control sample showed K_str_ = 0.58 and K = 0.05%, which indicated an unsatisfactory aggregate state of the soil and its instability to wind erosion according to the accepted criteria [89]. After the application of IPEC, the value of K_str_ increased to 2.30 units, and K increased 16-fold, qualifying the soil structure as excellent by existing standards. The treated soil layer with IPEC showed a twofold increase in the content of the agronomically valuable aggregate fraction (69.5%) compared to the control sample (35.4%).

The water stability criterion of soil aggregates W for the control sample was 80.5%, with the total amount of aggregates > 0.25 mm being 35.2% (satisfactory water stability). Upon IPEC treatment, the W criterion increased to 92%, and the total amount of aggregates > 0.25 mm increased to 69.5% (good water stability). Therefore, all the studied indicators demonstrated an improvement in the soil structure.

**Figure 9:**
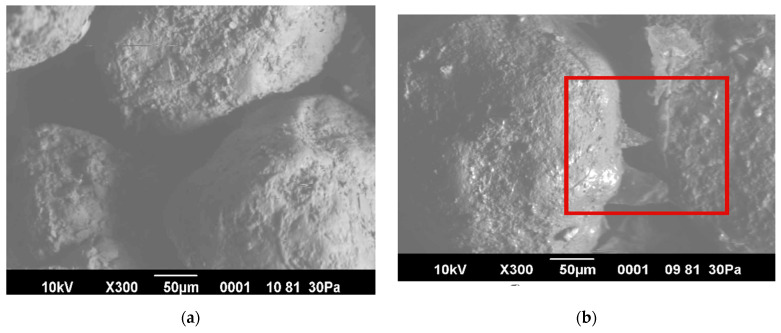
Microphotographs of soil particles: (**a**) after treatment with water and (**b**) after treatment with IPEC.

Figure 9 shows micrographs of the soil aggregates obtained after treatment with water and IPEC.

In Figure 9b, it can be seen that the ns-IPEC [Chitosan]:[Na-CMC] in a molar ratio of [3:7] covers the particles of unstructured pine sands and binds them into large aggregates at the particle contact points, forming a protective soil-polymer coating or crust. According to the authors [38], the resulting soil-polymer films do not block the pores in the soil layer, thus maintaining air and water exchange in the soil. Consequently, biodegradable polymers are effective for improving soil permeability, as they help create air pores in the soil and facilitate moisture penetration into the soil depth.

Figure S2 presents the results of the runoff of the control soil and soil treated with polymers and IPEC after water and wind erosion in a model experiment.

**Figure 10:**
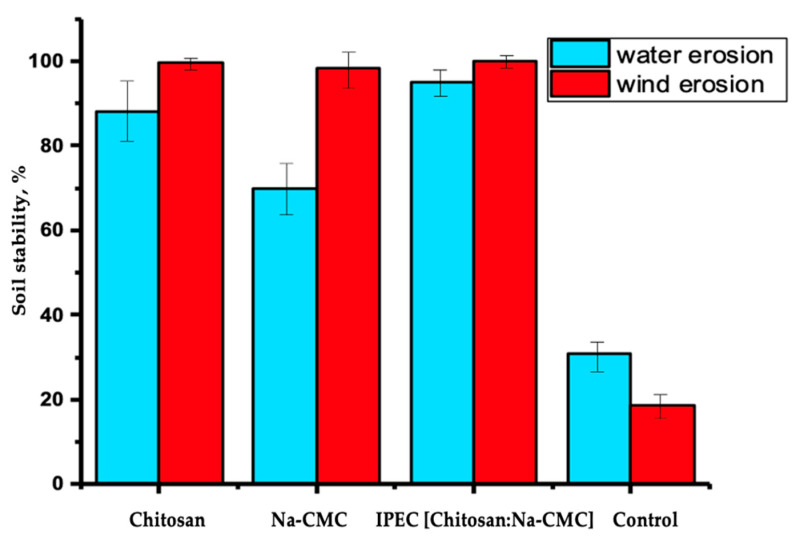
Resistance of soil samples to water and wind erosion.

The greatest soil surface erosion and runoff are observed in the control variant, while the least erosion is seen in the variants using polymers and IPEC. Based on the calculations of the mass of soil resistant to water and wind erosion, a diagram was created, as presented in Figure 10.

According to Figure 10, it can be asserted that the IPEC [Chitosan]:[Na-CMC] [3:7] demonstrates the highest soil stabilization effect against water and wind erosion. Post-IPEC treatment, soil resistance to water erosion increased by 64% and to wind erosion by 81%. The presence of hydrophilic (anionic) and hydrophobic (neutralized cation-anion) fragments in IPECs ensures electrostatic interaction with hydrophilic and hydrophobic sites on the soil particle surfaces, forming a protective polymer film on the soil surface or soil microaggregates [27,66,73,90]. 

**Figure 11:**
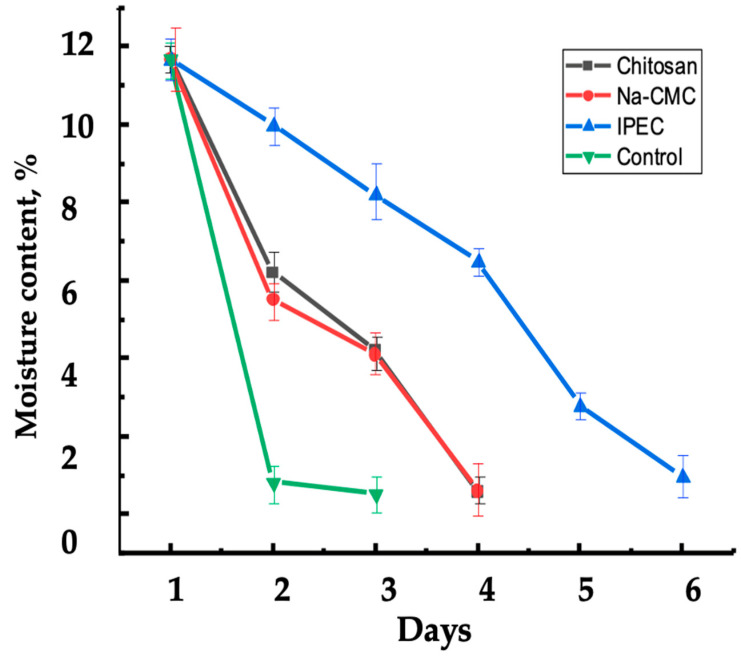
Dynamics of Moisture Retention in Soil Samples Treated with Polymers and IPEC over 6 Days.

This study showed that the best water retention effect for pine sands was observed with the application of IPEC. The initial moisture capacity of all soil samples was 11.68%. After 24 h, the water retention capacity of the soil in the control variant sharply decreased to 0.84%, while with the application of IPEC, it only slightly decreased to 9.9%. Thus, the use of IPEC retains soil moisture 9% more effectively per day and six times longer compared to the control variant (Figure 11). The application of IPEC promotes the formation of a porous soil structure, improving its water retention capacity. Additionally, the network structure of IPEC provides multiple absorption and controlled release of absorbed water, which enhances soil moisture retention and positively affects plant growth and development.

**Figure 12:**
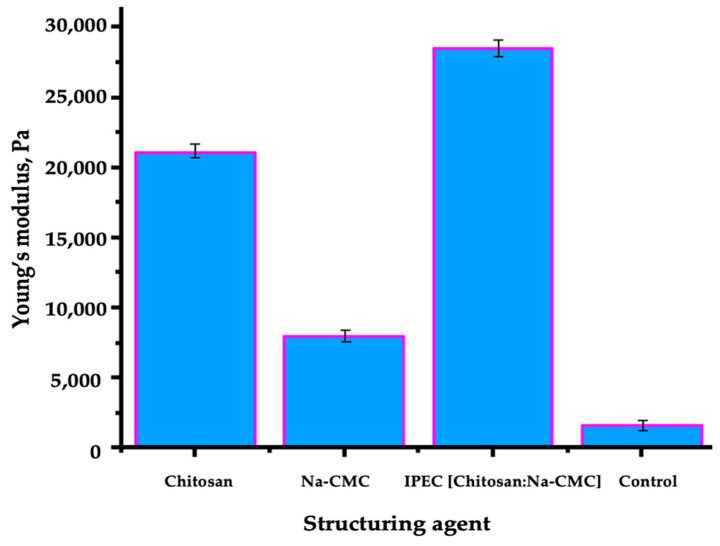
Mechanical Properties of Soil Aggregates.

Mechanical tests of soil-polymer coatings were conducted to demonstrate the impact of polymers and IPEC on the strength of the formed complexes and soil structure. The results of the study on the mechanical properties of soil structures are presented in Figure 12.

As shown in Figure 12, the Young’s modulus of the resulting films on the surface decreases in the following order: with IPEC—28,445 Pa, with chitosan—21,031 Pa, with Na-CMC—8000 Pa, and with distilled water—1619 Pa. The mechanical strength of structures based on IPEC was higher than that of structures treated with individual polymers and water. The established values of Young’s modulus are not critical for plants, as turgor pressure develops in plant cells as they grow [91].

Table 4 summarizes information on the strength of soil aggregates, their resistance to water and wind erosion, and the water retention capacity of various IPECs.

As illustrated in Table 4, the developed IPEC exhibits similar parameters for soil improvement and, in some cases, even demonstrates higher performance, particularly in water retention capacity. However, it is important to note the significantly lower mechanical strength of the samples, which is likely related to the adhesive properties of the polymers used. Thus, IPECs significantly enhance the mechanical strength of the soil without harmful effects on the environment and are promising soil structure formers. 

**Figure 13:**
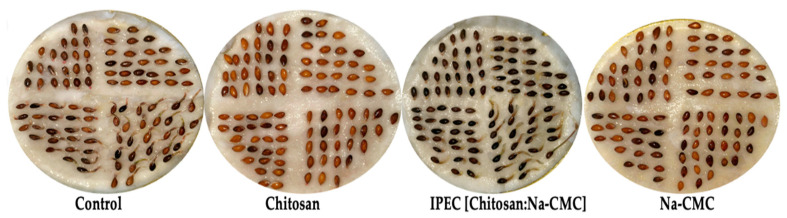
Scots pine seedlings treated with polymers and IPEC.

The study also examined the effect of polymers and IPEC on the sowing qualities of Scots pine seeds (Table 5, Figure 13).

From Table 5 and Figure 13, it is evident that the most effective treatment was with Na-CMC, where the seed germination energy reached 85% (12% higher compared to the control), and the germination rate reached 91%, corresponding to the first-class quality indicators. It was also observed that seedlings emerged 2 days earlier after Na-CMC treatment compared to control seedlings. The lowest biometric indicators were found with the use of chitosan and IPEC. This effect can be explained by the fact that the growth-stimulating action of chitosan and its IPEC is limited by various factors, including molecular weight, concentration, pH of the polymer solution, soaking time, and others. For example, in the study [95], a growth-stimulating effect of chitosan was observed at various concentrations and treatment durations for Ashwagandha (*Withania somnifera* L.) seeds. Additionally, according to the study by [96], chitosan encapsulation of seeds and its derivatives restores chlorophyll levels and photosynthesis in wilt-affected cotton plants (*Gossypium* L., 1753). Chitosan is an excellent biodegradable polymer film-former for hydrophobizing the surface and pelleting seeds. Hydrophobized seeds are protected from mold and pest damage. Pelleted seeds swell in moist soil and do not perish from a lack of moisture when the soil dries out. Encapsulation of seeds with chitosan and IPEC protects them from adverse environmental conditions and has an immunostimulatory effect. The elicitor activity of chitosan is due to the presence of N-acetylglucosamine residues in its molecule. The polycationic nature of chitosan allows it to bind to the negatively charged cytoplasmic membrane of bacterial cells through electrostatic interactions, inactivating the toxins of disease pathogens [97,98,99,100].

When treating the surface of pine sands in the forest seed complex of the Natural Reserve, ‘Semey Ormany’, polymers and IPECs facilitated the rapid germination of pine seeds and demonstrated better results compared to the control samples. For instance, when treated with the Na-CMC polymer, the first sprouts appeared on the fourth day, whereas in the control variant, they appeared on the seventh day. Additionally, there was an increase in germination energy, germination rate, uniformity, growth, needle count, and seedling biomass by 16–25% (Table S2, Figures S3 and S4).

Preliminary studies on the soil structuring properties of IPEC, conducted at the Natural Reserve “Semey Ormany” nursery, showed that in the control variant (Figure S4b), 40% of the soil was washed away during watering, whereas with IPEC application (Figure S3a), only 5% of the soil was washed away. IPEC increased soil structuring by 35%. Thus, the use of polymers and IPEC in forest cultivation production creates favorable conditions for seed germination and seedling growth, enhances soil biological activity, fertility, and water retention, and boosts plant immunity and stress resistance to drought, cold, and sharp temperature fluctuations.

## 4. Conclusions

The formation of n-IPEC through the interaction of chitosan and Na-CMC biopolymers with an optimal molar ratio of [Chitosan:Na-CMC] = [3:7] was confirmed by gravimetry, turbidimetry, and rheoviscometry methods. The stabilization of the complex is ensured by ion-ion interactions between macromolecules. Unlike the original polymers, the resulting IPEC offers several advantages, including good structuring and water retention properties, adjustable mechanical strength, biocompatibility, and insolubility in weakly acidic solutions. The application of IPEC to the soil also increased the concentrations of key agrochemical components, including humus by 17.89%, K₂O by 1.87%, Ca^2^⁺ by 2.58%, NH_4_⁺ by 29.45%, P_2_O_5_ by 12.79%, K⁺ by 10.15%, Na⁺ by 15.37%, Mg^2^⁺ by 16.74%, SO_4_^2−^ by 13.59%, and NO_3_^−^ content doubled.

Under laboratory conditions, the pre-sowing treatment of seeds with Na-CMC showed high germination results. Treatment with chitosan and IPEC contributes to seed encapsulation, which improves protection against pathogens and increases drought resistance. As a result of the vegetation experiment conducted in the nursery, after the application of IPEC to the soil surface, an increase in seed germination energy by 15%, seed germination rate by 14%, shoot length by 0.5 cm, root length by 2.5 cm, stem diameter by 0.08 cm, the average number of needles by 12, and seedling biomass by 0.43 g was observed.

These results confirm the effectiveness of IPEC [Chitosan: Na-CMC] as a soil structuring agent for forest sandy soils and a biostimulator for Scots pine seed growth.

## Figures and Tables

**Table 1:** Examples of the use of polymers for improving the quality of forest soils.

Polymer	Soil Type	Result	Reference
Epoxidized linseed oil + wastes	Two types of forest soils: deciduous and coniferous	The tested biocomposites demonstrated promising potential for application as mulching films in agriculture or forestry as ecofriendly alternatives to polyethylene films.	[28]
Acrylic acid/Acrylamide based superabsorbent polymers (SAP)	Sandy soils	The performance of SAPs depends on the pore space and a saline environment in the soil, and low SAP application rates are suitable for maximizing the water available to plants in sandy soils.	[29]
PAM	Calcareous montmorillonitic sandy clay loam, non-calcareous kaolinitic sandy loam	The application of 25 and 50 kg ha^−1^ of granular PAM reduced soil erosion by 23 and 57%, respectively, compared to the untreated control.	[30]
TerraCottem arbor^®^ PAM-free water absorbent polymer	Calcisol with a sandy loam texture	The benefits of the new soil conditioner were highest when applied at doses of 40 or 80 g per seedling.	[31]
PAM/polyacrylic acid salts	Carbonate Loamy–Sandy Arenosol from the Emirate of Dubai Loamy–Sandy Retisol from the Moscow Region, Russia Loamy Serozem from the Tashkent Region, Uzbekistan	Gel-forming polymer conditioners and new technologies of their application increase the productivity of plant crops and the quality of biomass by 30–50%, with a 1.3–2-fold saving of water resources and reliable protection of the topsoil from pathogens and secondary salinization.	[32]

**Table 2:** Agrochemical indicators of the surface layer (0–25 cm) of pine sands.

№	Agrochemical Parameters	Control	IPEC [Chitosan]:[Na-CMC] [3:7]
1	pH_H2O_	6.1	6.0
2	pH_KCl_	6.5	6.3
3	Hygroscopic moisture, %	1.86	2.23
4	Humus, %	0.95	1.12
5	NO_3_ content, mg/kg	1.45	3.50
6	P_2_O_5_ content, mg/kg	12.90	14.55
7	K_2_O content, mg/kg	90.80	92.50
8	Salinity, mg/kg		
8.1	K^+^	22.26	24.52
8.2	Na^+^	20.03	23.42
8.3	NH_4_^+^	2.75	3.56
8.4	Mg^2+^	8.84	10.32
8.5	Ca^2+^	32.59	33.43
8.6	Cl^−^	1.15	1.16
8.7	SO_4_^2−^	10.30	11.70

**Table 3:** Content of mechanical element fractions of different sizes in forest sandy soil.

Options	Control	IPEC [Chitosan]:[Na-CMC] [3:7]
k air-dry		
>10 mm	0	0
10–0.25 mm	35.4	69.5
<0.25 mm	61.2	30.2
water-stable		
>1 mm	0.2	0.6
>0.25 mm	35.4	
indicators of soil structure		
K_str_	0.58	2.30
K, %	0.05	0.80
W, %	80.5	92.0

**Table 4:** The impact of various interpolyelectrolyte complexes (IPECs) on the characteristics of soil aggregates.

IPEC	Type of Soil	Mechanical Strength	Wind Erosion Resistance	Water Erosion Resistance	Increasing of Water Retention Capacity	Reference
HYPAN- PDADMAC *	Sand Soil	16 MPa 10MPa	100% 100%	60% 99%	1.5 time	[92]
ALG-QHECE **	Sand Soil	30 MPa 165 MPa	- -	90% 99%	- -	[38]
BSM-PDADMAC ***	Soil	0.8–45 MPa	-	100%		[93]
Chitosan-Sodium alginate	Dark chestnut soil	70 kPa	-	97%	-	[72]
PDADMAC- HYPAN	Sand	19 MPa	-	90%	-	[94]
Chitosan- Na-CMC	Sandy soil	28 kPa	>99%	97%	6 time	Present work

* HYPAN—hydrolyzed polyacrylonitrile, PDADMAC—poly (N, N′-diallyl-N, N′-dimethylammonium chloride, ** ALG—sodium alginate, QHECE—quaternized hydroxyethyl cellulose ethoxylate, *** BSM—butadiene-styrene microspheres.

**Table 5:** Effect of polymers and IPEC on the germination of Scots pine seeds.

Options	Germinated Seeds, pcs (Total of 100 pcs)				Germination Energy, %	Germination Rate, %	Uniformity, %	Root Length, mm
	Days							
	5	7	10	15				
Chitosan	0	6	6	3	12	15	1.5	0.10 ± 0.02
Na-CMC	4	70	11	6	85	91	9.1	0.40 ± 0.05
IPEC	0	2	5	7	7	14	1.4	0.10 ± 0.03
Control	0	60	13	2	73	75	7.5	0.30 ± 0.05

## Data Availability

The original contributions presented in the study are included in the article/Supplementary Materials, further inquiries can be directed to the corresponding author/s.

## Supplementary Materials

The following supporting information can be downloaded at: https://www.mdpi.com/article/10.3390/polym16162373/s1, Figure S1: Precipitates of Synthesized IPEC [Chitosan]:[Na-CMC] at Different Molar Ratios [0:10], [1:9], [2:8], [3:7], [4:6], [5:5], [6:4], [7:3], [8:2], [9:1], and [10:0]: (a) Wet IPEC Precipitate, (b) Dry IPEC Precipitate, Figure S2: Surface of soil samples after water and wind erosion experiment, Figure S3: Effect of polymers on the morphological and biometric indicators of pine seedlings. (a) control (b) chitosan (c) Na-CMC (d) IPEC [Chitosan:Na-CMC], Figure S4: Application of IPEC as a soil structuring agent in the nursery: (a) pine seedlings grown after soil treatment with IPEC, (b) control pine seedlings grown on untreated soil; Table S1: Physico-chemical characteristics of used polymers, Table S2: Results of studies on the treatment of soils with polymers and IPECs on the growth and development of Scots pine (*Pinus sylvestris* L.) seeds and seedlings.
