# Supplementary material for: Development of Interpolyelectrolyte Complex Based on Chitosan and Carboxymethylcellulose for Stabilizing Sandy Soil and Stimulating Vegetation of Scots Pine (Pinus sylvestris L.)
Source: Polymers (Basel). 2024 Aug 22;16(16):2373. doi: 10.3390/polym16162373 (PMC11359870; doi:10.3390/polym16162373)
Supplement: Supplementary file 1 [file polymers-16-02373-s001.zip › polymers-3127888-supplementary.pdf]

Development of Interpolyelectrolyte Complex Based on Chitosan and Carboxymethylcellulose for Stabilizing Sandy Soil and Stimulating Vegetation of Scots Pine (*Pinus sylvestris* L.)

Nazira Berikbol, Alexey Klivenko, Vadim Markin, Lazzyat Orazzhanova, Gulnur Yelemessova and Zhanar Kassymova

Supplementary Information

Table S1. Physico-chemical characteristics of used polymers

Polymers	MM, kDa	S, %	*DS,* %	Degree of substitution	CMG %	pH
Chitosan	60	86.4	83.9	-	-	4.2
Na-CMC	92	98.0	-	0.88	26.4%,	6.7

Table S2. Results of studies on the treatment of soils with polymers and IPECs on the growth and development of Scots pine (*Pinus sylvestris L.)* seeds and seedlings.

Options	Germination Energy, %	Germination Rate, %	Average shoot length (above-ground), mm	Average root length (below-ground), mm	Average stem diameter, mm	Number of needles, pcs	Average needle length, cm,	Average seedling biomass, g
Chitosan	80	80	7.0	6,8	2,00	53	2.52	1.14
Na-CMC	90	91	7.0	6,7	2,00	57	2.40	1.13
IPEC	85	89	6.5	6,5	2,00	57	2.20	1.13
Control	70	75	6.0	4,0	1,92	45	2.00	0.70

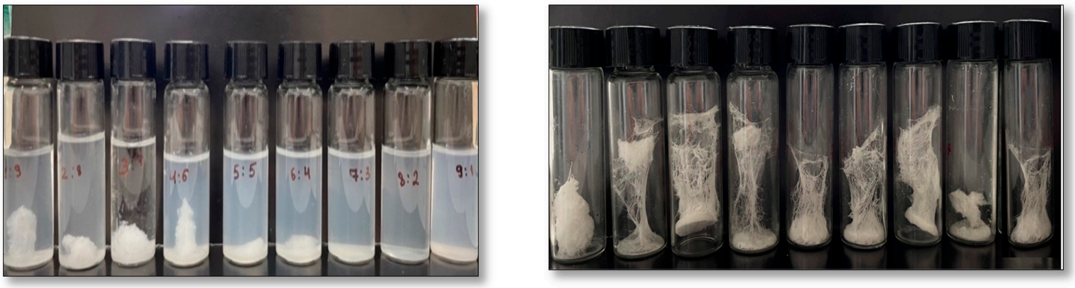

(**a**) (**b**)

Figure S1. Precipitates of Synthesized IPEC [Chitosan]: [Na-CMC] at Different Molar Ratios [0:10], [1:9], [2:8], [3:7], [4:6], [5:5], [6:4], [7:3], [8:2], [9:1], and [10:0]: (a) Wet IPEC Precipitate, (b) Dry IPEC Precipitate.

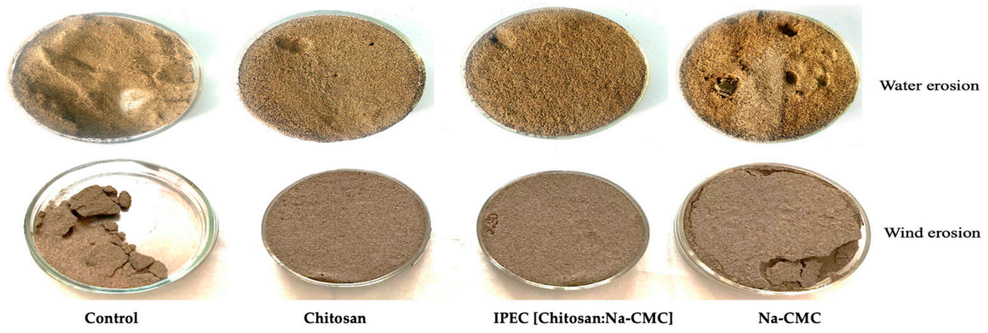

Figure S2. Surface of soil samples after water and wind erosion experiment.

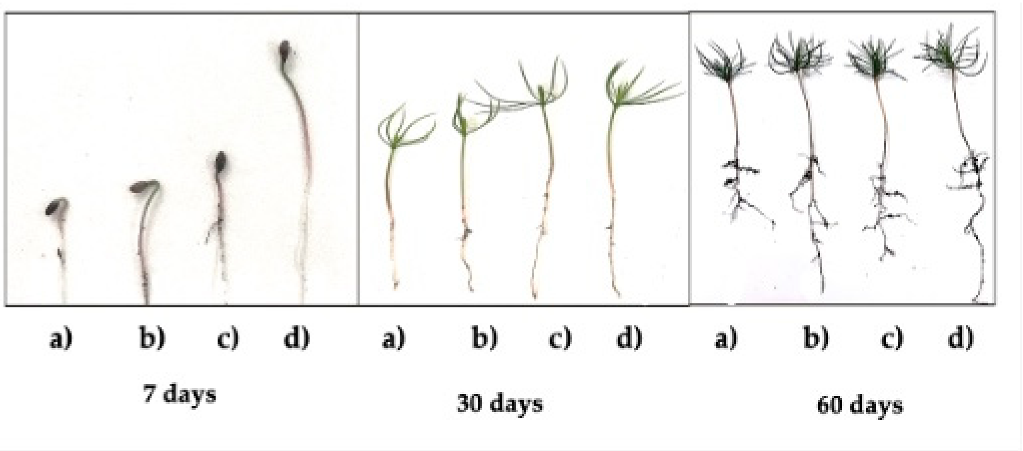

Figure S3. Effect of polymers on the morphological and biometric indicators of pine seedlings. a)control b) chitosan c) Na-CMC d) IPEC [Chitosan:Na-CMC]

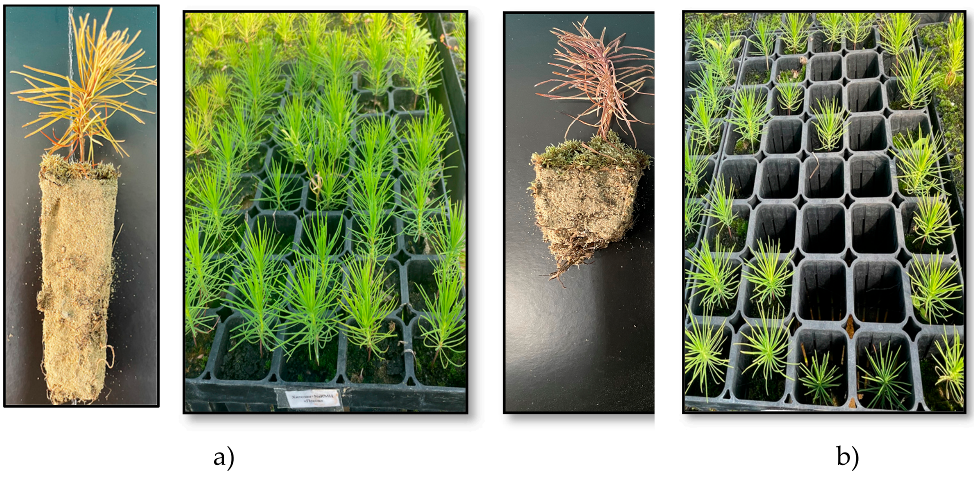

Figure S4. Application of IPEC as a soil structuring agent in the nursery: a) pine seedlings grown after soil treatment with IPEC, b) control pine seedlings grown on untreated soil.
